# Spinal Epidural Abscess: A Single-Center Retrospective Review of Incidence, Risk Factors, and Management at a Community Hospital

**DOI:** 10.7759/cureus.82727

**Published:** 2025-04-21

**Authors:** Tyler E Rice-Canetto, Louis Reier, Mohammad Arshad, Yasir R Khan, Emilio C Tayag

**Affiliations:** 1 Neurosurgery, Arrowhead Regional Medical Center, Colton, USA; 2 Neurosurgery, California University of Science and Medicine, Colton, USA; 3 Neurosurgery, Riverside University Health System Medical Center, Moreno Valley, USA; 4 Neurosurgery, Desert Regional Medical Center, Palm Springs, USA; 5 Neurology and Neurosurgery, Desert Regional Medical Center, Palm Springs, USA

**Keywords:** differential diagnosis of spinal epidural abscess, spinal epidural abscess, spinal epidural abscess decompression, spinal epidural abscess diagnosis, treatment of spinal epidural abscess

## Abstract

Introduction: While relatively rare, spinal epidural abscess (SEA) is a neurosurgical pathology that can result in life-altering spinal cord injury if not properly managed or if diagnosis is delayed. The textbook presentation of SEA includes the triad of back pain, fever, and neurologic deficits; however, this classic triad is infrequently seen. Due to the nonspecific nature of each individual symptom, the condition is often misdiagnosed, resulting in delayed management and potentially significant consequences for the patient. This study aims to characterize the incidence, risk factors, and management of patients presenting with SEA at Desert Regional Medical Center (DRMC) to optimize the timely identification and treatment of affected individuals at our California community hospital.

Materials and methods: We conducted a single-center retrospective review at DRMC of patients diagnosed with SEA. The data collection period spanned from July 1, 2016, to April 29, 2021, and included a total of 88 patients. For each patient, we extracted data on demographics, risk factors, comorbidities, clinical presentation, diagnostics, management, and discharge disposition. We generated frequency tables for qualitative data and conducted exploratory data analysis for quantitative variables, accompanied by a series of visualizations to illustrate our findings. To assess the suitability of parametric testing for future studies, we created Q-Q plots and performed Shapiro-Wilk and Kolmogorov-Smirnov tests.

Results: Our study population demonstrated a male predominance, with a mean age of 57 years. Among the risk factors and comorbidities evaluated, recent infection, smoking, alcohol use, and diabetes mellitus were the most prevalent. Only 6% (5) of patients presented with the classic triad of back pain, fever, and neurologic deficits. The most common locations of epidural abscesses included the lumbar spine, lumbar plus sacral spine, and thoracic spine. Bacteremia was present in 81% (71) of patients, and biopsy cultures were positive in 77% (68) of those who underwent the procedure. The most common treatment approach was a combination of surgical intervention and antibiotic therapy tailored to culture results.

Conclusions: This study enabled us to characterize the SEA patient population at our institution and compare it to the broader literature, facilitating timely diagnosis and management. Given the condition's tendency to present with nonspecific symptoms and elevated but unremarkable inflammatory markers, this additional data may help reduce diagnostic delays and improve patient outcomes. Beyond its implications for SEA management within the DRMC neurosurgical department, this study contributes to the existing body of literature, supporting improved diagnostic and therapeutic strategies at other institutions as well.

## Introduction

Spinal epidural abscess (SEA) is a suppurative infection within the spinal epidural space that can lead to spinal cord injury from mechanical compression and/or vascular occlusion [1]. The infection most commonly develops through indirect hematogenous spread, but it may also result from direct inoculation [2]. Commonly cited risk factors include diabetes mellitus, IV drug use (IVDU), immunodeficiency, hepatic and renal disease, recent spinal surgery with use of hardware, vascular catheters, osteomyelitis-discitis, and other infections [3,4].

The classic presentation of SEA is a triad of localized back or neck pain, fever, and focal neurological deficit. However, this combination of symptoms is only seen at initial presentation in 8-15% of cases [4,5]. This, combined with the nonspecific nature of these symptoms, delays diagnosis in approximately 89% of patients [4,5]. Other disease states that may present similarly and thus delay diagnosis of SEA include osteomyelitis-discitis, spondylolisthesis, malignancy, metastatic cancer to the spine, psoas and/or other soft tissue abscess, epidural hematoma, and phlegmon [6]. In some cases, SEA is a neurosurgical emergency, and if left untreated, it can lead to permanent neurological deficits such as paralysis or death from septicemia [7]. Although SEA is relatively rare, its incidence has increased from 0.2 to 1.2 per 10,000 hospital admissions in the 1970s to present-day figures of 5.1-8 per 10,000 hospital admissions [8,9]. This increase in incidence is attributed to an aging population and its accompanying comorbidities, an increase in the number of spinal surgeries with instrumentation, higher rates of IVDU, and advancements in imaging [2,10].

The workup for suspected SEA includes a complete blood cell count (CBC) with differential, inflammatory markers such as erythrocyte sedimentation rate (ESR), C-reactive protein (CRP), and procalcitonin (Procal), blood cultures, and radiographic imaging [4,11,12]. Elevated laboratory values, the presence of bacteremia, and positive imaging findings each increase the likelihood of SEA diagnosis. Gadolinium-enhanced magnetic resonance imaging (MRI) is the gold standard imaging modality, with a sensitivity and specificity of greater than 90% [7,13]. T1-weighted images with contrast typically show a hypointense lesion (representing the abscess) with peripheral enhancement, often in a circumferential pattern [13]. Abscesses will be hyperintense on T2-weighted imaging [13]. Finally, diffusion-weighted imaging typically shows restricted diffusion [14]. If MRI is contraindicated or unavailable, computed tomography (CT) with myelography can be used as an alternative. A potential complication that providers must consider with CT myelography is the risk of introducing infection into the subarachnoid space, which can spread throughout the entire central nervous system [15].

The management of SEA typically involves a combination of medical and surgical interventions. Upon suspicion of the diagnosis, providers will begin broad-spectrum antibiotics in all patients. The determination, then, of whether a patient is a surgical candidate is complex and provider-driven, but one common indicator is an acute neurological deficit attributable to the infection [16]. Much of the current literature aims to determine who can be managed medically and who should undergo surgery among those who are neurologically intact at initial presentation. For both non-surgical and surgical patients, the provider will obtain a culture of the abscess, either via interventional radiology (IR) or surgery [11]. Once the causative organism is identified, a regimen of tailored antibiotic therapy can be started [11]. A standard antibiotic regimen typically includes IV administration for four to six weeks, followed by oral administration for two to four weeks (or eight weeks if concurrent osteomyelitis is present) [10]. Blood cultures can also be used to tailor antibiotic therapy when abscess cultures lack growth, due to the high concordance of SEA causing bacteremia [17]. For patients who are surgical candidates, intervention typically consists of decompressive laminectomy with washout of the infection [10]. For high-risk candidates, CT-guided fine-needle aspiration via IR is an option. According to current management protocols, the estimated mortality rate ranges from 3.7% to 5% [5].

The purpose of this study is to evaluate the incidence, risk factors, and management of SEA at our local community hospital. Doing so will enable us to understand the disease burden in our community better and investigate which factors put our patients at the highest risk of developing this infection. The intention is to implement these findings hospital-wide, to speed up the diagnosis of SEA in affected patients and thereby minimize delays in management, ultimately optimizing patient outcomes. Our study will also contribute to the existing body of literature on SEA, increasing the availability of information on diagnosis for other providers and facilities.

## Materials and methods

Study design

We performed a retrospective review of patients admitted to Desert Regional Medical Center (DRMC) with the diagnosis of spine epidural abscess. DRMC is a 387-bed tertiary acute care community hospital located within Riverside County, in Palm Springs, California. It is a Level II trauma center, certified as a stroke center and a certified STEMI (ST-elevation myocardial infarction) center, with 70,000 emergency room visits annually.

Inclusion and Exclusion Criteria

The inclusion criteria consisted of adults aged 18 years or older who were admitted to DRMC between July 1, 2016, and April 29, 2021. Eligible patients had an initial diagnosis of SEA based on clinical status and MRI findings, which was subsequently confirmed by surgical or IR cultures.

Data Collection

MetroWest Medical Center Institutional Review Board (IRB) approval was obtained (approval number: 2021-051). A list of patients admitted to DRMC between July 1, 2016, and April 29, 2021, with an ICD-10 code of G06.1 (intraspinal abscess and granuloma) who were at least 18 years of age was requested. This initial list consisted of 276 patients. We removed any patients who had a diagnosis other than SEA, including 41 patients with a diagnosis of brain abscess and an additional 34 patients with diagnoses including prevertebral abscess, osteomyelitis/discitis, or paraspinal abscess. From the remaining 201 patients, we filtered out duplicate medical record numbers, resulting in 93 patients. Finally, five patients were excluded who were diagnosed at outside hospitals and had never been seen by our neurosurgical department, leaving a total of 88 patients to be included in our study. For each patient, we extracted the following variables: sex, age, race, new vs previously diagnosed status, body mass index (BMI), chief complaint (back or neck pain, fever/malaise, weakness, other), abscess location (cervical, thoracic, lumbar, sacral), concurrent osteomyelitis/discitis, other abscess, diabetes mellitus, IVDU, alcohol consumption, tobacco use, recent known infection and type, sacral decubitus ulcer or equivalent, immunocompromised state and cause if present (cancer, HIV, other), other pertinent past medical history, laboratory markers of infection/inflammation (white blood cell count (WBC), ESR, CRP, Procal), presence of bacteremia and organism if so, treatment (surgical vs. medical and type), pathology from biopsy if relevant, and disposition.

Statistical analysis

We conducted a simple statistical analysis on the data extracted. For qualitative data with binary values, we created frequency tables with relative percentages, some of which are provided in the results section. These tables provided the foundation for organizing our data and assessing potential imbalances within our sample, while also helping readers quickly digest data for a variable of interest. For several of the qualitative variables, we created bar graphs and pie charts to illustrate the relative frequencies and percentages within each category. For our quantitative data, we conducted exploratory data analysis (EDA) to help summarize our findings and determine the appropriateness of subsequent parametric testing. Our quantitative variables include age, BMI, WBC, ESR, CRP, and Procal. Our EDA started with basic descriptive statistics, eliciting means, medians, modes, and ranges. We then created a series of visualizations, including histograms, box plots, and quantile-quantile (Q-Q) plots, for each variable included in the results section of this paper. Each histogram is a visual representation of the relative frequencies within a given category. Overlying each histogram is a kernel density estimation (KDE), a curve used to apply our sample data to estimate a probability distribution for the population. The box and whisker plots help represent the first quartile (Q1), the second quartile (Q2), the third quartile (Q3), and the fourth quartile (Q4), as well as any outliers in the data. The “box” represents the interquartile range (IQR), which is the middle 50% of the data, and the “whiskers” extend to the minimum and maximum that are not outliers. Lastly, the Q-Q plots show how the data distribution from our patients deviates from a normal distribution. For the quantitative variables where a standard normal distribution of the data was not evident, we conducted subsequent Shapiro-Wilk and Kolmogorov-Smirnov tests to confirm. These tests, in conjunction with the Q-Q plots, dictate the appropriateness of using parametric testing as a next step in future publications. Parametric testing is suitable when the assumption of normality is met, while non-parametric testing is appropriate when this assumption is not met.

## Results

Demographics

**Figure 1:**
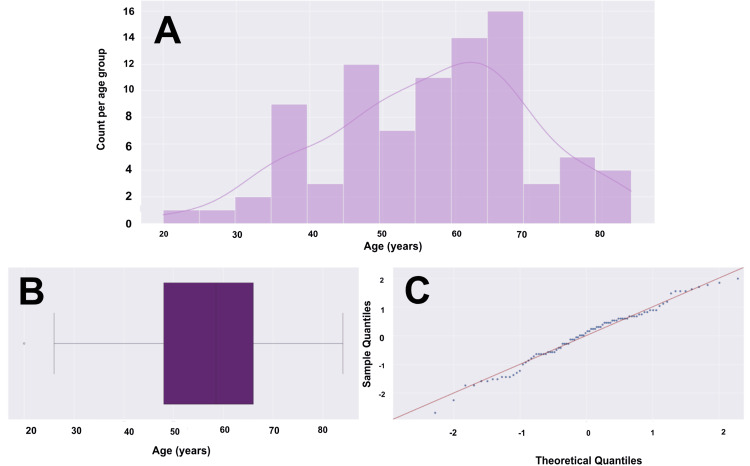
Age histogram, box plot, and Q-Q plot for a cohort of 88 patients (A) Histogram with overlying KDE. (B) Box and whisker plot with the left whisker representing the 0th percentile at age 26, and the right whisker representing the 100th percentile at age 84, excluding any outliers. The box represents the IQR, with the left boundary of the box being the 25th percentile (Q1) at age 48 and the right boundary of the box being the 75th percentile (Q3) at age 65. This means that the middle 50% of patients fall within the age range of 48 to 65 years. The vertical line within the IQR box represents the median (Q2), at age 58. There is one outlier to the left, at the age of 20. Including the one outlier, the data range is 64 years. (C) Q-Q plot with the blue dots representing our age data and the red line representing a standard normal distribution. We are confident in a normalized distribution of standardized data based on visualization. Our age data will likely hold up in parametric testing. Q-Q: quantile-quantile, KDE: kernel density estimate, IQR: interquartile range

Our study included a total of 88 patients, 68 of whom (77.3%) were male and 20 (22.7%) were female. The mean age was 56.81 years, with a range of 20 to 84 years. Figure 1 provides additional data on age within our cohort, including statistical descriptions in the legend. Notably, 57 (64.8%) identified as Caucasian individuals, and 21 (23.9%) identified as Hispanic individuals. The remaining 10 (11.3%) were comprised of African American, Filipino, Middle Eastern, and Samoan individuals, with one patient of unspecified race.

Clinical characteristics (comorbidities and associated characteristics)

**Figure 2:**
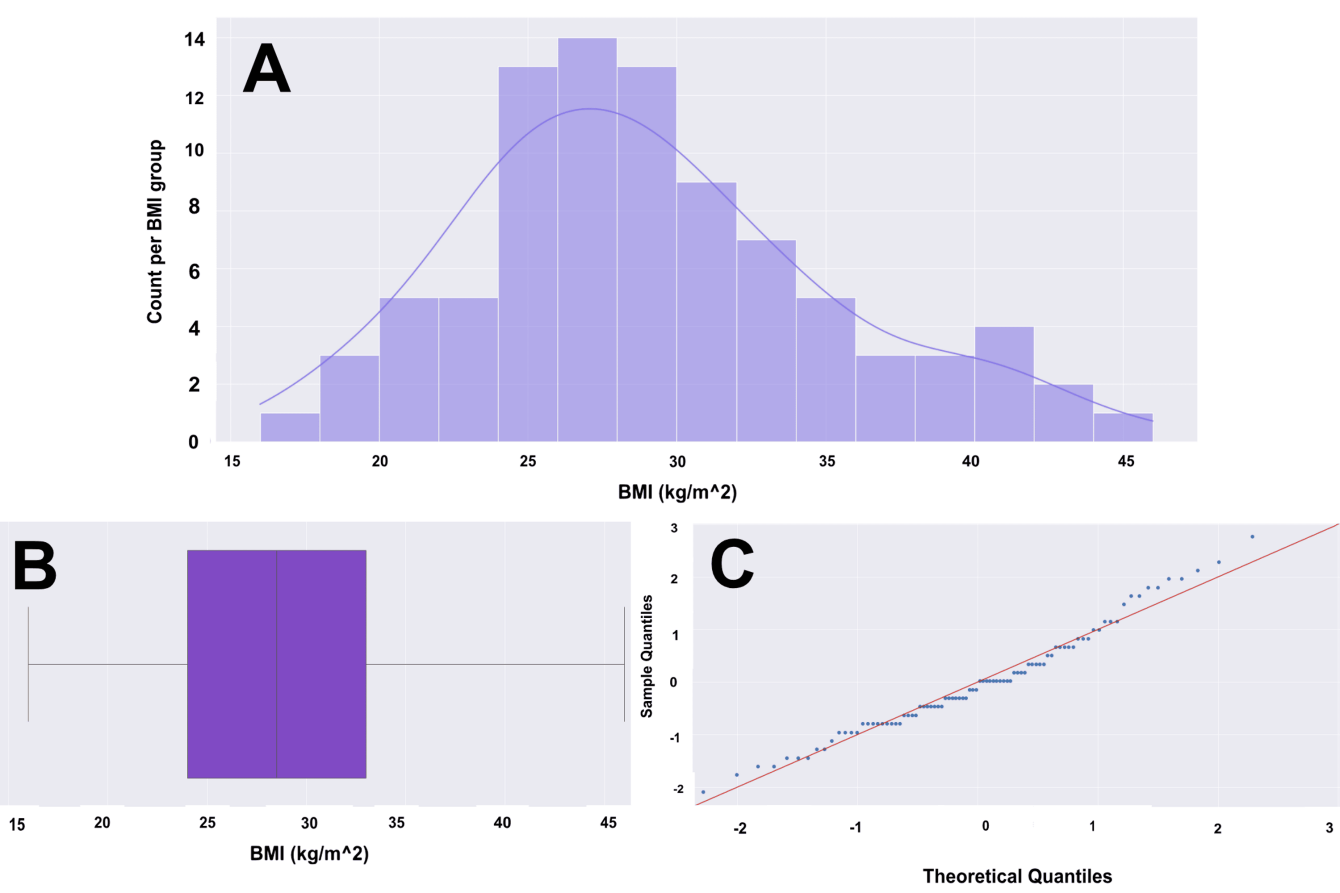
BMI histogram, box plot, and Q-Q plot for a cohort of 88 patients (A) Histogram with overlying KDE. (B) Box and whisker plot with the left whisker representing the 0th percentile at a BMI of 16, and the right whisker representing the 100th percentile at a BMI of 46. The box represents the IQR, with the left boundary of the box being the 25th percentile (Q1) at a BMI of 24 and the right boundary of the box being the 75th percentile (Q3) at a BMI of 33. This means that the middle 50% of patients have a BMI between 24 and 33. The vertical line within the IQR box represents the median (Q2), at a BMI of 28. The BMI data does not have any outliers. The data has a range of 30 units. (C) Q-Q plot with the blue dots representing our age data and the red line representing a standard normal distribution. We are confident in a normalized distribution of standardized data based on visualization. Our BMI data will likely hold up in parametric testing. BMI: body mass index, Q-Q: quantile-quantile, KDE: kernel density estimate, IQR: interquartile range, kg: kilograms, m^2: meters squared

We examined a series of comorbidities within our cohort. The average BMI of our patients was 29, with a range of 16 to 46, as shown in Figure 2. Twenty-nine (33%) had diabetes mellitus, 41 (47%) were either current or former smokers, 33 (38%) had a history of alcohol use, and 25 (29%) had a history of IVDU. We also examined several known risk factors for SEA, including immunodeficiency, hepatic and renal disease, concurrent osteomyelitis or discitis, and recent infection. For immunodeficiency, we looked at rates of cancer and HIV specifically, with five (5.7%) with each diagnosis. When assessing conditions such as hepatic and renal disease, 15 (17%) had a past medical history, including chronic kidney disease and/or cirrhosis, and nine (10.2%) had a history of hepatitis C. Concurrent osteomyelitis or discitis was present in approximately 58 (66%) of cases. Lastly, we had a slight predominance of patients who identified a recent infection, accounting for 46 (52.3%) of our cases.

Seventy-eight patients (89%) were diagnosed with SEA at our facility, while 10 (11%) had a previously established diagnosis. In terms of abscess location within the spinal column, 21 (24%) had abscesses located solely in the lumbar spine, and 21 (24%) had abscesses located only in the thoracic spine. Twenty (22.7%) had epidural abscesses in both the lumbar and sacral regions. Ten (11.5%) had an abscess isolated to the cervical spine, while the remaining 16 (18%) had abscesses in other combinations of locations, as shown in Table 1.

**Table 1:** SEA location frequencies and percentages for a cohort of 88 patients N: number (frequency), %: relative percentage, SEA: spinal epidural abscess

Location	Number of subjects (N, %)
Lumbar	21 (24%)
Thoracic	21 (24%)
Lumbar, sacral	20 (23%)
Cervical	10 (11.5%)
Thoracic, lumbar	7 (8%)
Cervical, thoracic	5 (6%)
Thoracic, lumbar, sacral	2 (2%)
Cervical, lumbar	1 (1%)
Cervical, lumbar, thoracic	1 (1%)

Regarding symptoms, 34 patients (39%) presented with only back or neck pain, and 21 (24%) presented with back pain and weakness. Table 2 demonstrates the different combinations of presenting symptoms.

**Table 2:** SEA presenting complaint frequencies and percentages for a cohort of 88 patients N: number (frequency), %: relative percentage, SEA: spinal epidural abscess

Presenting complaint	Number of subjects (N, %)
Back or neck pain	34 (38.5%)
Back pain with weakness	21 (24%)
Other	10 (11.5%)
Back pain with fever/malaise	9 (10%)
Weakness	5 (6%)
Back pain with weakness and fever	5 (6%)
Fever/malaise	3 (3.5%)
Fever with weakness	1 (1%)

Fifty-eight patients (65%) had a concurrent diagnosis of osteomyelitis, and 60 (68%) had an additional abscess located somewhere outside of their spine. Approximately 46 (53%) had a known recent infection. Seven (8%) had a history of abscesses or multiple abscesses in other parts of their body, five (6%) had a recent lung infection, and an additional five (6%) had a history of osteomyelitis or discitis in the extremities. Four (4%) had a urinary tract infection, and an additional four (4%) had an infection from a foreign object or a post-surgical infection.

Seven (8%) had a history of sacral decubitus ulcers or an equivalent. A total of 10 (12%) had an immunocompromised state, five (6%) had HIV, and the other five (6%) had cancer. In addition to the associated conditions previously stated, nine (10%) had hepatitis C, and eight (9%) had cardiac diseases. Forty-six (52.5%) had no other contributing conditions.

Diagnostics and management

**Figure 3:**
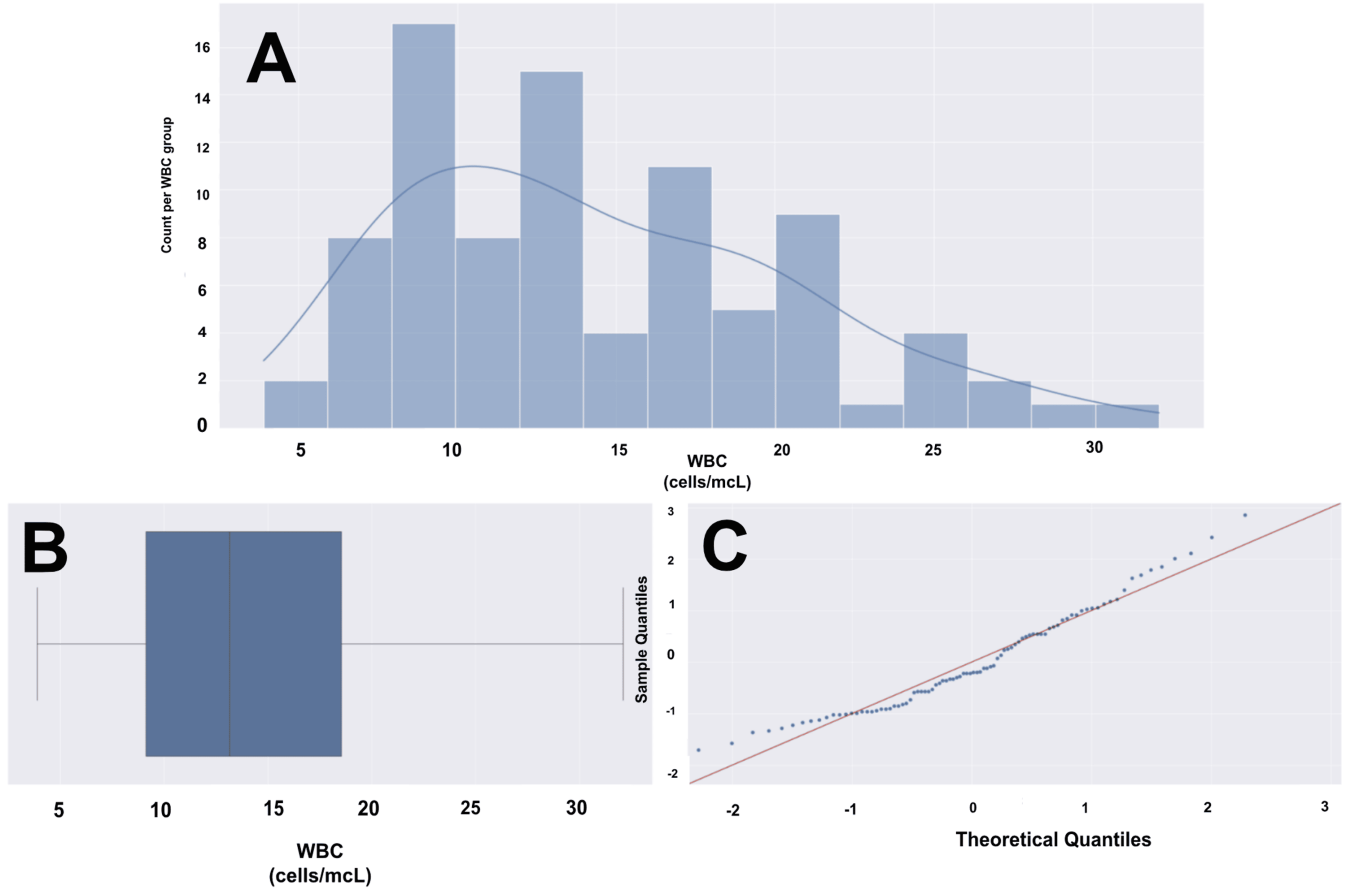
WBC histogram, box plot, and Q-Q plot for a cohort of 88 patients (A) Histogram with overlying KDE. (B) Box and whisker plot with the left whisker representing the 0th percentile with a WBC of 4, and the right whisker representing the 100th percentile with a WBC of 32. The box represents the IQR, with the left boundary of the box being the 25th percentile (Q1) with a WBC of 9 and the right boundary of the box being the 75th percentile (Q3) with a WBC of 18. This means that the middle 50% of patients have a WBC between 9 and 18. The vertical line within the IQR box represents the median (Q2), with a WBC of 13. The WBC data does not have any outliers. The data has a range of 28 units. (C) Q-Q plot with the blue dots representing our age data and the red line representing a standard normal distribution. We are not confident in the normalized distribution of standardized data based on the visualization. The Shapiro-Wilk test showed a significant departure from normality (sig = 0.96, p = 0.004), and the Kolmogorov-Smirnov test showed a significant departure from normality (sig = 0.998, p = 1.64). WBC data would not hold up in parametric testing. WBC: white blood cell count, Q-Q: quantile-quantile, KDE: kernel density estimate, mcL: microliter, IQR: interquartile range

**Figure 4:**
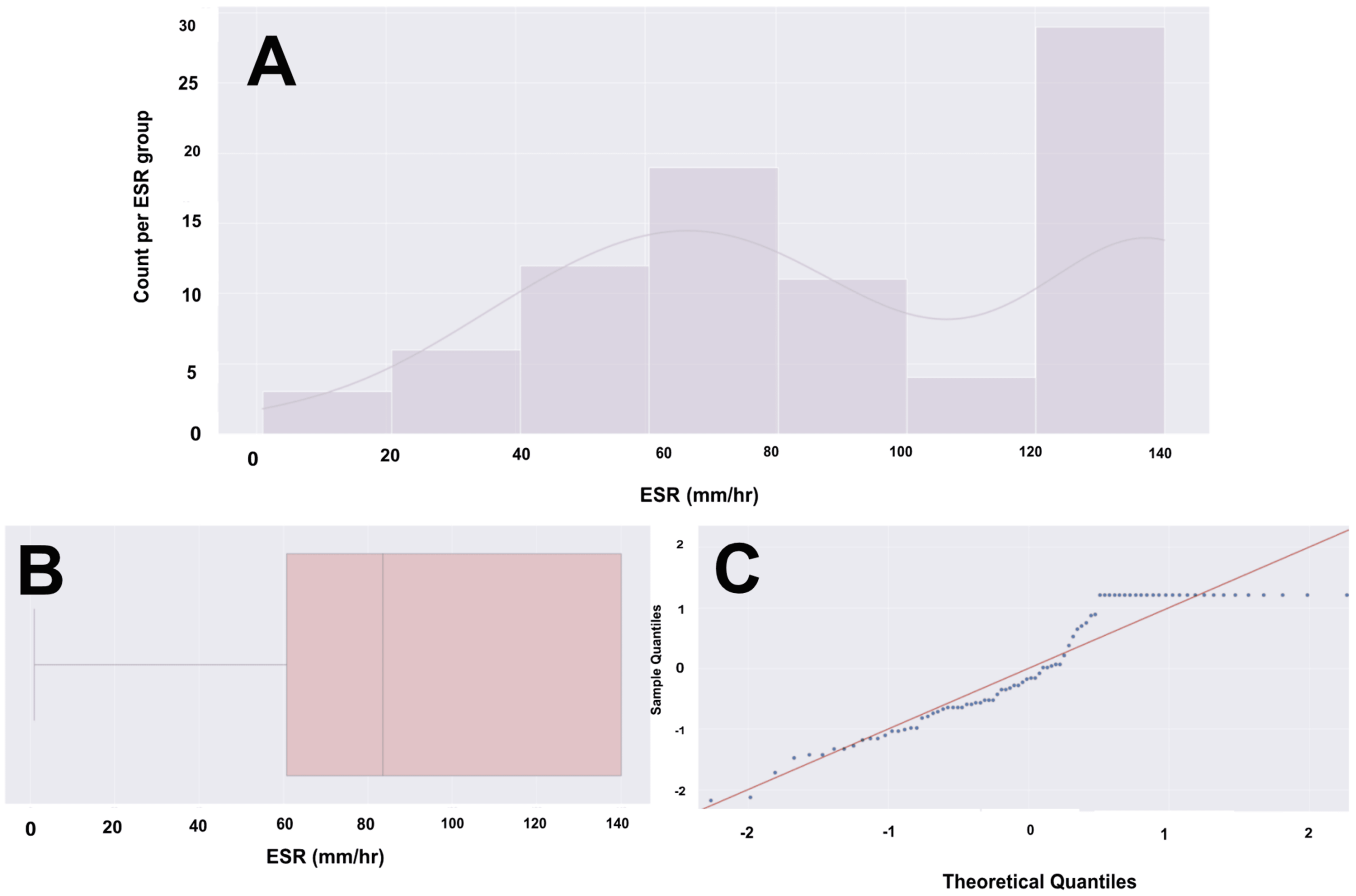
ESR histogram, box plot, and Q-Q plot for a cohort of 88 patients (A) Histogram with overlying KDE, representing a bimodal distribution. (B) Box and whisker plot with the left whisker representing the 0th percentile with an ESR of 0, and the right whisker representing the 100th percentile with an ESR of 140. The box represents the IQR, with the left boundary of the box being the 25th percentile (Q1) with an ESR of 60 and the right boundary of the box being the 75th percentile (Q3) with an ESR of 140. In this case, the Q3 and Q4 are the same value of 140, as the high number of patients with elevated ESR values shifted the data towards the maximum. The vertical line within the IQR box represents the median (Q2), with an ESR of 83. The ESR data does not have any outliers. The data has a range of 139 units. (C) Q-Q plot with the blue dots representing our age data and the red line representing a standard normal distribution. We are confident, based on the visualization, that there is a significant departure from normality. ESR data would likely not hold up in parametric testing. ESR: erythrocyte sedimentation rate, Q-Q: quantile-quantile, KDE: kernel density estimate, IQR: interquartile range, mm: millimeters, hr: hour

**Figure 5:**
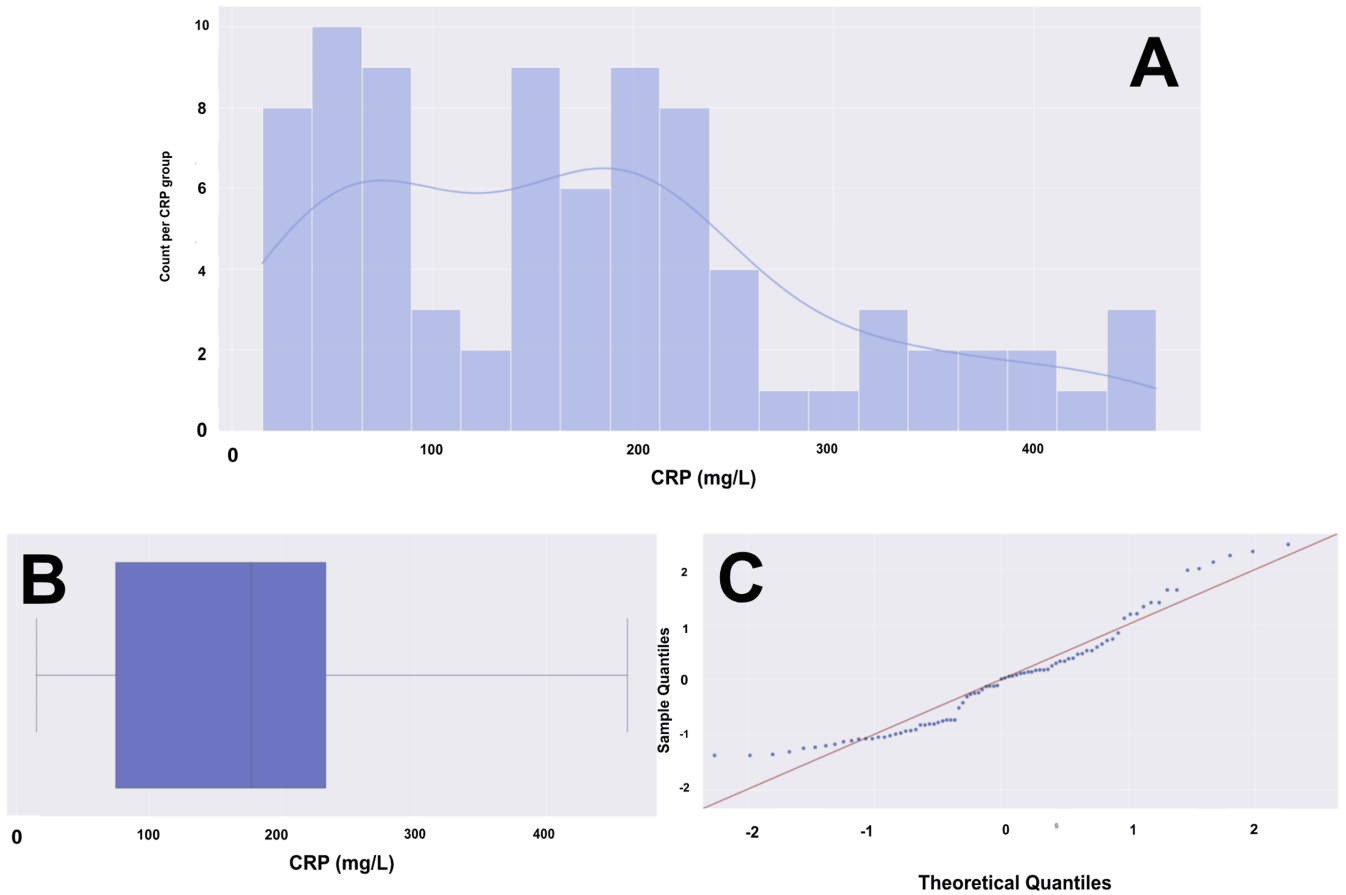
CRP histogram, box plot, and Q-Q plot for a cohort of 88 patients (A) Histogram with overlying KDE. (B) Box and whisker plot with the left whisker representing the 0th percentile with a CRP of 15, and the right whisker representing the 100th percentile with a CRP of 461. The box represents the IQR, with the left boundary of the box being the 25th percentile (Q1) with a CRP of approximately 80 and the right boundary of the box being the 75th percentile (Q3) with a CRP of approximately 225. This means that the middle 50% of patients fall between a CRP of approximately 80 and 225. The vertical line within the IQR box represents the median (Q2), with a CRP of approximately 180. The CRP data does not have any outliers. The data has a range of 447 units. (C) Q-Q plot with the blue dots representing our age data and the red line representing a standard normal distribution. We are not confident in a normalized distribution of standardized data based on visualization. The Shapiro-Wilk test showed a significant departure from normality (sig = 0.94, p = 0.0004), and the Kolmogorov-Smirnov test showed a significant departure from normality (sig = 0.993, p = 1.15). CRP data would not hold up in parametric testing. CRP: C-reactive protein, Q-Q: quantile-quantile, KDE: kernel density estimate, IQR: interquartile range, mg: milligram, L: liter

**Figure 6:**
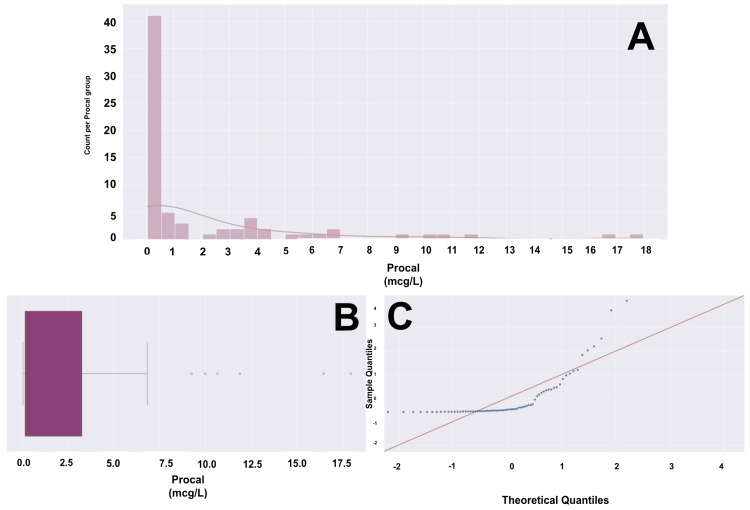
Procal histogram, box plot, and Q-Q plot for a cohort of 88 patients (A) Histogram with overlying KDE. (B) Box and whisker plot with the left whisker representing the 0th percentile with a Procal of approximately 0, and the right whisker representing the 100th percentile with a Procal of approximately 7. The box represents the IQR, with the left boundary of the box being the 25th percentile (Q1), and a Procal of approximately 0.1, and the right boundary of the box being the 75th percentile (Q3), with a Procal of approximately 2.75. This means that the middle 50% of patients fall between a Procal of approximately 0.1 and 2.75. The vertical line within the IQR box represents the median (Q2), with a Procal of approximately 0.3. The Procal data has 6 outliers, at values of approximately 9, 10, 11, 12, 16, and 18. The data has a range of 18 units. (C) Q-Q plot with the blue dots representing our age data and the red line representing a standard normal distribution. We are confident, based on the visualization, that there is a significant departure from normality. Procal data would likely not hold up in parametric testing. Procal: procalcitonin, Q-Q: quantile-quantile, KDE: kernel density estimate, IQR: interquartile range, mcg: microgram, L: liter

Laboratory tests (WBC, ESR, CRP, and Procal) were obtained for all of our patients. Figures 3-6 provide visualizations of the data for each collected lab value. The mean values for WBC, ESR, CRP, and Procal were 14.43, 90.24, 175.12, and 2.27, respectively. Fifty-three patients (60%) had elevated WBCs, and 81 (92%) had an elevated ESR, with three (3%) for whom an ESR was not obtained. Eighty-three (94%) had elevated CRP, and this result was not collected in a total of five (6%). Lastly, 32 (36%) had an elevated Procal, with results not collected in 17 (19%).

**Figure 7:**
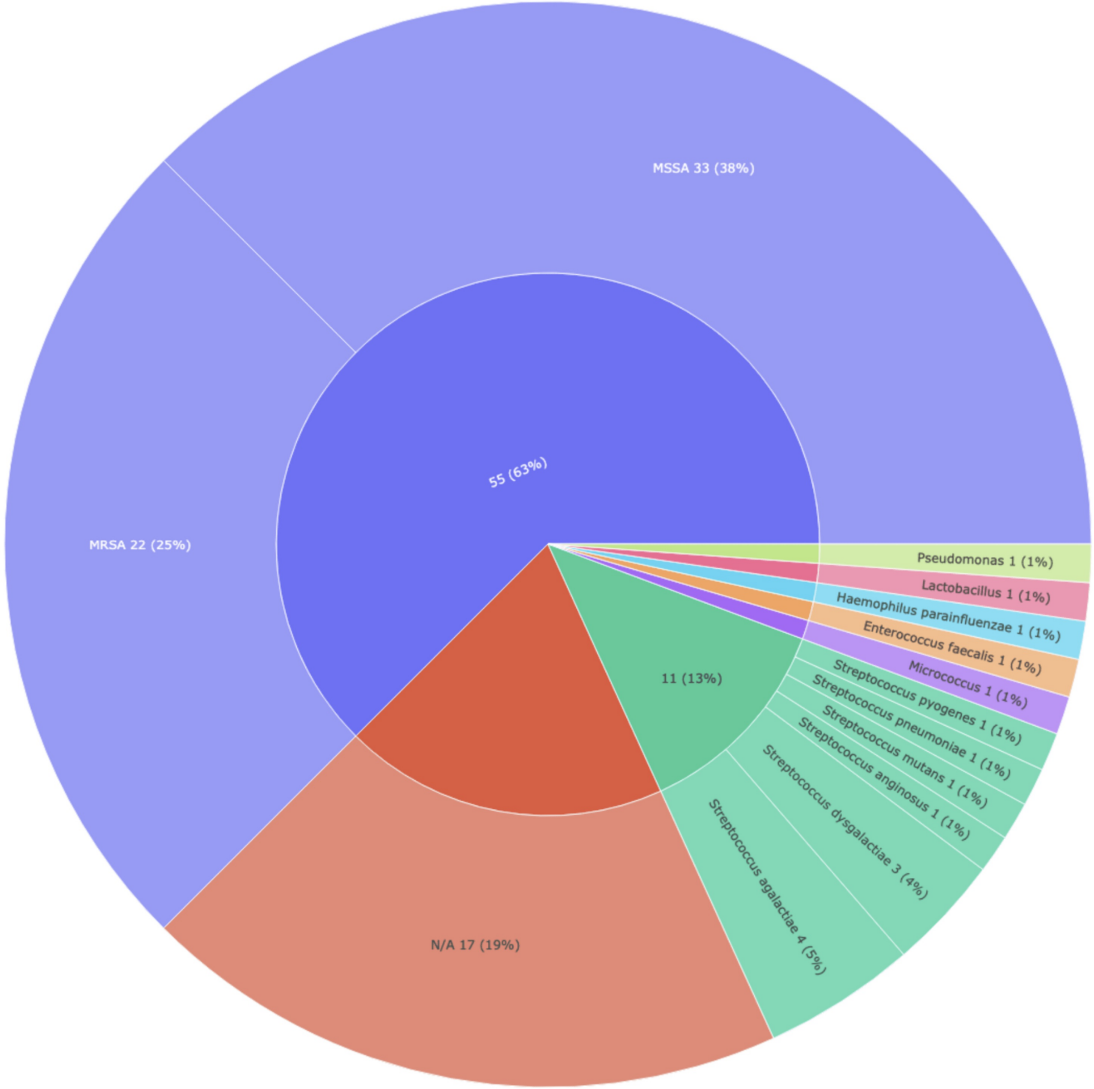
SEA blood culture species pie chart for a cohort of 88 patients In this pie chart, the width of each slice represents the relative frequency of a given organism. Each color group species is within the same genus. N/A is representative of patients who did not have any growth on blood cultures. Next to each category is the quantitative data in the format "number of patients (percentage of patients)". MRSA: methicillin-resistant *Staphylococcus aureus, *MSSA: methicillin-sensitive *Staphylococcus aureus, *N/A: not applicable, SEA: spinal epidural abscess

Seventy-one patients (81%) had positive blood cultures. Of those, 33 (46.5%) had methicillin-susceptible *Staphylococcus aureus* (MSSA) and 22 (31%) had methicillin-resistant *Staphylococcus aureus* (MRSA). Figure 7 provides a visualization of the organisms implicated in our cohort’s blood culture results. A majority of our patients underwent a biopsy of the epidural abscess, with only 11 (13%) without specimens. Twenty-four (28%) were identified as MSSA and 17 (20%) as MRSA. Twenty (23%) had a biopsy culture that did not show growth of any organism.

**Figure 8:**
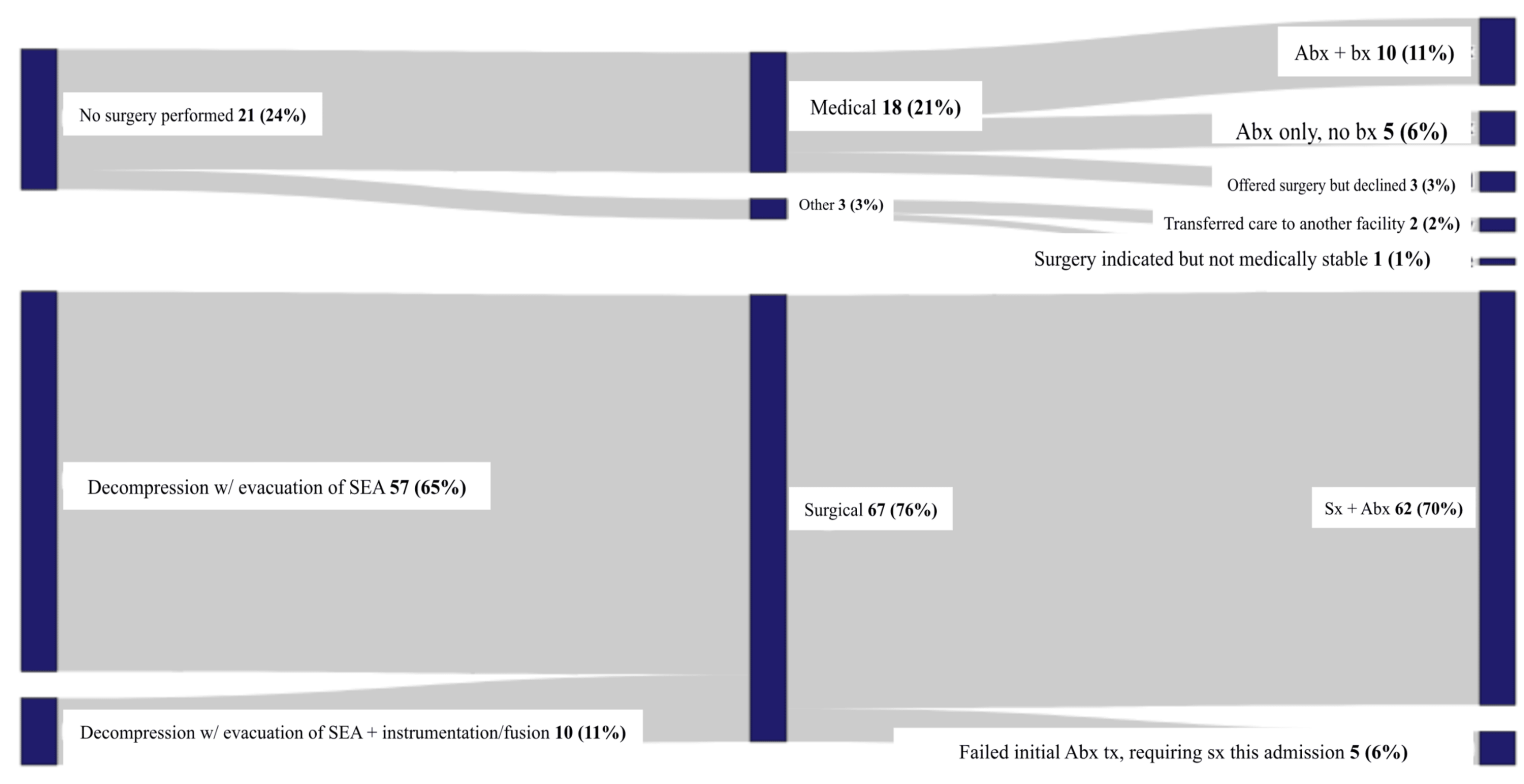
Treatment Sankey diagram for a cohort of 88 patients The Sankey diagram represents two categories of treatment, medical and surgical, with the details branching off for each. The vertical size of each segment represents the relative frequency of a given management. Next to each category is the quantitative data in the format "number of patients (percentage of patients)". SEA: spinal epidural abscess, Abx: antibiotics, Bx: biopsy, Sx: surgery

Frequencies of treatment method, medical or surgical, are shown in Table 3. Figure 8 provides a flow chart of the different interventions within these categories. Sixty-two (70%) had their SEA treated via surgery and antibiotics, and 10 (11%) underwent biopsy and received antibiotics. Five (6%) only required antibiotic management, while an additional five (6%) failed initial antibiotic treatment and required surgery during the current admission. Sixty-seven (76%) underwent some form of surgical intervention. Fifty-seven (65%) had decompression only with evacuation of the spinal abscess, while 10 (11%) had decompression and evacuation of the abscess with accompanying instrumentation/fusion for stabilization.

**Table 3:** SEA treatment for a cohort of 88 patients N: number (frequency), %: relative percentage, SEA: spinal epidural abscess

Treatment	Number of subjects (N, %)
Surgery with antibiotics	62 (70.5%)
Antibiotics with biopsy	10 (11.5%)
Failed initial antibiotic treatment, requiring surgery	5 (6%)
Antibiotics only	5 (6%)
Patient offered surgery but declined.	3 (3.5%)
Transferred to higher care	2 (2.5%)
Not stable for surgery but offered if gets medically stable	1 (1%)

**Figure 9:**
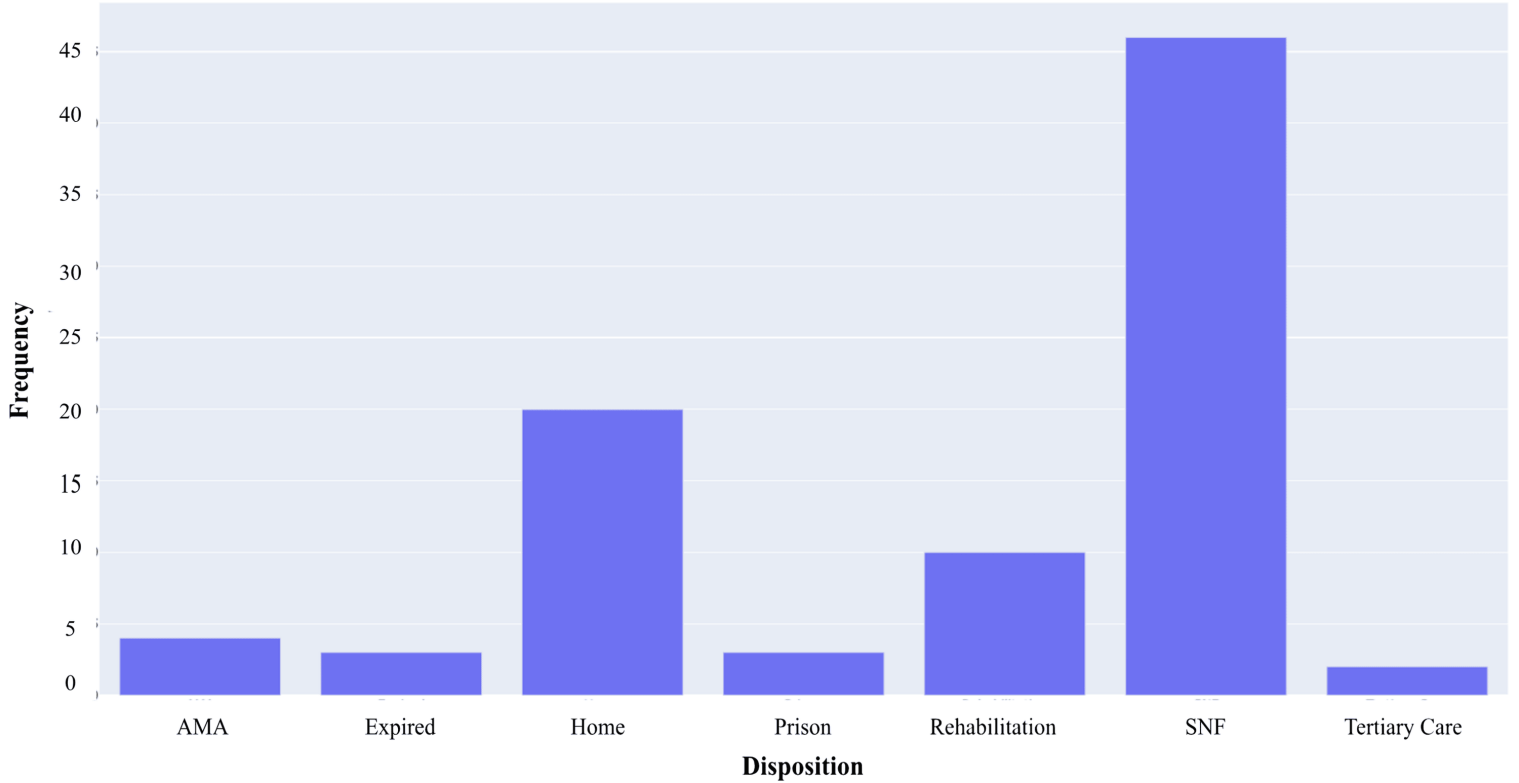
SEA disposition bar plot for a cohort of 88 patients This bar plot shows each discharge type and its corresponding frequency. AMA: against medical advice, SNF: skilled nursing facility, SEA: spinal epidural abscess

Data on patient disposition is revealed in Figure 9. Twenty patients (23%) were discharged home, and four (5%) went home against medical advice. Forty-six (52%) were discharged to a skilled nursing facility, and 10 (11%) were sent to inpatient rehabilitation. Three (3%) went to prison following treatment, and an additional three (3%) passed away while inpatient. Lastly, two (2%) were transferred to a higher-level care hospital due to complex medical conditions unrelated to their SEA.

## Discussion

The primary purpose of this study was to assess the incidence, risk factors, and management of patients presenting with SEA to our community hospital. Doing so enables us to identify population-specific risk factors and understand the disease burden within our community. To better understand what makes our patient population unique, we compared our results to those in the general population by extracting similar figures from the current literature.

Regarding our patient demographics, the male-to-female ratio of 3.4:1 exceeds the range documented in the literature, which typically ranges from 2.1 to 3.1 [18]. Our patient population’s mean age of approximately 57 years is consistent with the literature’s figure for those presenting with SEA, which is between the fifth and sixth decades of life [3,18]. For race, the distribution at hospitals nationwide is similar to that of the United States as a whole, with approximately 19% of the population being Hispanic individuals and 81% non-Hispanic individuals [3,9]. For our racial data, the Hispanic population accounted for nearly 24% of SEA patients, with the remaining racial groups comprising 76%. Our hospital’s higher proportion of Hispanic patients compared to national data is presumably attributable to the higher percentage of Hispanic individuals in Riverside County, as cited by the census at nearly 52% [19].

In terms of the different clinical characteristics that we extracted data on, we assessed various patient risk factors and presented symptoms. Within the literature, diabetes mellitus and IVDU are listed as the two most significant risk factors for the development of SEA [4]. Other cited risk factors that we examined include IVDU and alcohol use. Within our data set, relative incidences of these risk factors from highest to lowest were smoking at 47%, alcohol consumption at nearly 38%, diabetes mellitus at 33%, and IVDU at 29%. We also assessed known risk factors, such as immunodeficiency, hepatic disease, renal disease, osteomyelitis or discitis, and other recent infections [20]. Of all these, the highest relative incidence was among patients with a recent infection, accounting for 52.3% of cases. Identifying each patient’s risk factors for SEA is extremely valuable in helping to guide diagnosis and prognosis [4,11].

In addition to analyzing risk factors, we assessed the patient’s chief complaint and compared it to the literature. The classic triad of localized neck or back pain, fever (identified as fever or malaise in our study), and focal neurological deficit (identified as weakness in our study) was present in only 5.7% of cases. The literature also cites a relatively low incidence of this constellation of symptoms, ranging from 8% to 15% [4,5]. These low figures, combined with the nonspecific nature of each symptom in the triad, help explain why diagnosis may be delayed in up to 89% of cases [5]. Many of our patients presented with only one or two of these symptoms, and 11.4% presented with something different. Additionally, 23.9% presented with back pain and weakness but no fever, highlighting the need for clinicians to include inflammatory markers in their laboratory panels when patients present with refractory neck or back pain and accompanying neurological deficits [21]. Lastly, in terms of abscess location, 59% had a lesion localized to a single segment of the spinal column, specifically the cervical, thoracic, or lumbar region. Overall, whether in isolation or combination with another vertebral region, the incidence of abscesses in each location, from highest to lowest, was lumbar, thoracic, sacral, and cervical. Understanding the relative incidences of symptoms and abscess locations within the spinal column may help clinicians determine the likelihood of SEA in a patient’s presentation when formulating their differential diagnoses.

First-line diagnostics for patients suspected of having SEA included radiographic imaging and laboratory tests. Indicated laboratory tests, including a CBC with differential, inflammatory markers (ESR, CRP, Procal), and blood cultures, were assessed for each patient [12]. A biopsy of the abscess was subsequently obtained as needed, either via IR or surgery. Starting with the WBC count from the CBC, 60% of patients had an elevated count (>12), and 48 of those patients had lab values greater than 12.5, a value associated with an increased likelihood of SEA [11]. Examining ESR, the most sensitive and specific of the inflammatory markers, 93% had values greater than 20 mm/hr, a value concerning for the presence of an abscess [4]. A CRP value greater than 115 is also shown in the literature to be associated with an increased likelihood of SEA, and this was the case in 60% of our patients [11]. Thirty-six percent had an elevated Procal, which, when examined in conjunction with ESR, helps monitor clinical progression and efficacy of treatment [22]. The presence of bacteremia is also associated with an increased likelihood of SEA and was present in 81% of our patients [4]. Approximately 88% of our patients underwent diagnostic biopsy, 23% of which had no organism growth on cultures. For those with positive cultures, nearly 28% of specimens grew MSSA, and almost 20% grew MRSA. This data is consistent with the literature, which indicates that *Staphylococcus aureus* is the most commonly implicated organism, accounting for roughly 66% of all cases [1,11,12,16].

Management of SEA for this study was categorized based on whether it involved medical or surgical interventions. Roughly 21% of patients only underwent medical management, three of whom were offered surgery but declined. These patients received antibiotic regimens tailored to their blood culture results or biopsy results, if available, as recommended in the literature [11,17]. The retrospective review by Patel et al. identified four key predictors of medical failure: diabetes mellitus, a CRP level greater than 115 mg/L, a WBC count greater than 12.5 × 10^9/L, and positive blood cultures. The presence of one or more of these increases a patient's risk of failing medical management by anywhere between 35.4% and 76.9%, while those with none of these risk factors have only an 8.3% chance of treatment failure [11]. Examining our patient data, we found that 67% of patients had diabetes, 53% had a qualifying ESR, 56% had a qualifying WBC count, and 81% had positive blood cultures. These factors helped guide clinical decision-making regarding whether to pursue medical or surgical intervention at our institution. Approximately 76% of our patients underwent surgery (either with or without instrumentation/fusion), accompanied by antibiotic therapy. The literature suggests that posterior fixation should be considered in cases of significant vertebral dislocation, often in conjunction with vertebral osteomyelitis [21]. Nearly 66% of our cohort had osteomyelitis/discitis, and just over 10% of our patients underwent instrumentation. This confirms that vertebral osteomyelitis is a consideration for this surgical intervention, based on severity and other factors, but it is not an absolute indication for it.

While this study is a significant first step in characterizing our hospital’s SEA patient population and understanding the disease burden in our community, further analyses are needed to quantify the relative risk and assess correlations. Future studies could examine the relationship between different demographics, comorbidities, and lab values and their impact on management and outcome. Next steps would include conducting further statistical analyses to determine the presence of a correlation between all these variables and the relative correlation coefficients of each variable with the likelihood of having SEA. Further study could also help assess the social determinants of health that impact the management and outcomes of this condition at our hospital. We can evaluate whether two racial groups, for example, are two distinct populations in terms of management and outcomes. This study and future studies have immense potential to help improve the timely diagnosis of SEA and guide management, optimizing patient outcomes, both at our community hospital and elsewhere.

Study limitations

This retrospective cohort study was limited by its analysis of SEA cases at a single center, resulting in a relatively small sample size of 88 patients. Further studies examining similar variables in other hospitals within Riverside County, as well as nationally or even internationally, are worthwhile to increase the scope of this research. Such follow-up studies would also help evaluate clinical decision-making and implement quality improvement measures within the neurosurgical department at DRMC. Additionally, the fact that this was a retrospective study limited our data collection, as we were confined to variables of interest that had already been collected from patients. Another limitation of our study was storing and categorizing data by variable of interest rather than by patient. While doing so helped ensure patient de-identification and simplify exploratory data analysis, for this study, our capacity for more advanced statistical testing was limited. For future publications, we would like to reorganize the data by patient instead of by variable to enable comparative statistical testing. This would require renewing our IRB request to reaccess patient records. Next steps would then include using our assessments of whether our data is normally distributed, as discussed in the methods and results section, to conduct more intensive statistical analysis. This may include methods such as the Student's t-test for parametric variables or the Mann-Whitney U test for non-parametric variables. This type of analysis will enable us to evaluate the impact of social determinants of health, such as those on patient management and outcomes.

Despite these limitations, our study enabled us to characterize the population of patients presenting to DRMC with a diagnosis of SEA. Through our research, we have summarized the incidence, risk factors, diagnostics, management, and disposition of our patient base. Having a greater understanding of how this condition presents and progresses in our population will help support our providers and trainees as they assess and manage patients.

## Conclusions

Analyzing each of these variables in patients with a SEA at DRMC, compared to the literature, provides a stronger understanding of the demographics and risk factors in our presenting population. Having a sense of our hospital’s SEA population will provide one more piece of information to our providers as they develop their differential diagnosis, which will help reduce delays in diagnosis and intervention. Comparing our management data to the literature is also extremely valuable for assessing our hospital’s standards of care and the appropriateness of medical versus surgical intervention for SEA. Our neurosurgical department will use our findings to minimize adverse patient outcomes, including failed medical management and unnecessary surgical procedures. In addition to the value this research brings to our community hospital, it contributes to the existing body of work on SEA and guides diagnostics and management at other institutions.
